# The Hospital. Nursing Section

**Published:** 1901-12-07

**Authors:** 


### The Hospital.

### Nursing Section.

Contributions for this Section of "The Hospital" should be addressed to the Editor, "The Hospital" Nursing Section, 28 & 29 Southampton Street, Strand, London, W.C.

No. 793.—Vol. XXXI. *SATURDAY, DECEMBER 7, 1901.*

### Notes on News from the Nursing World.

### PENSION FUND SOUVENIR.

The proprietors of The Hospital beg to announce that Messrs. Marion, of Soho Square, have kindly offered to supply frames, in brown or green wood with bevelled gilt edges, to contain the Souvenir plate at the exceptionally low price of 2s. 6d. If nurses who have already subscribed will send this further remittance, a frame in the colour of their choice will be forwarded, packing and postage free, to their address so long as it is within the United Kingdom. Intending subscribers can order the plate framed complete for the same additional sum of 2s. 6d. Messrs. Marion have had complete charge of the production of the album and plate, and the proprietors of The Hospital feel sure that the nurses of the Royal National Pension Fund will appreciate their excellent work. They have spared no trouble in the undertaking, and the offer of these frames shows a continuation of their generous interest.

### OUR CLOTHING DISTRIBUTION.

We have to acknowledge the receipt of further parcels of useful clothing from our readers as follows:—"A small contribution to The Hospital clothing distribution, from Policy 101"; Nurse Cecil, 15 Farm Avenue, Streatham, who mentions the interesting fact that all the articles she sends were, with one exception, voluntary contributions from patients; and Miss Helen Gafton, Wessington Court, Woolhope, Hereford.

### NURSES AND THE BENEVOLENT FUND.

So far, we are sorry to hear, the shilling subscriptions for the Junius S. Morgan Benevolent Fund are not nearly equal in number to those received in 1900. It is probable that many nurses have overlooked the matter, and we therefore remind them that in order that their subscriptions may be included in this year's work, they should send their shillings in at once if possible, and certainly before the 31st instant, to Mr. Louis H. M. Dick, Secretary, Royal National Pension Fund for Nurses, 28 Finsbury Pavement, E.C.

### THE ROYAL RED CROSS.

The following notification has been issued by the War Office:—"In view of the investiture which His Majesty the King proposes to hold on December 17th, those ladies now in this country who have been appointed to the Order of the Royal Red Cross for services in South Africa, and who have not received the decoration, are requested to communicate at once by letter with the Under-Secretary of State for War, if they have not already done so."

### WAR NURSES.

The *Orient* and the *Lake Erie* arrived at Southampton on Monday, the former having on board Nursing Sisters J. Clay, L. Clarke, and A. F. Allerman (all returning to South Africa); and the latter Nursing Sisters M. Glenton-Kerr and A. Spooner. Miss Glenton-Kerr returns to South Africa after a month's leave and Miss Spooner is relinquishing army duty. The *Dilwara* left Capetown on November 28 with the following nursing sisters on board:—M. J. Burdett, L. Warriner, L. L. Lovett, D. J. Richards, and M. S. Bidmead.

### NURSES FROM INDIA.

The *Plassey* arrived at Southampton from India on Saturday with the following nursing sisters on board:—A.N.S. Superintendent Sister Mackay (from Cairo, on leave); Sister Stannistreet, I.A.N.S. (on leave); and Lady Superintendent Moore, I.A.N.S. (on one year's leave).

### A NEW NURSES' HOME FOR ST. THOMAS'S HOSPITAL.

It has become absolutely requisite for the authorities of St. Thomas's Hospital to raise a sum of £50,000 in order to erect a new nurses' home. In 1871, when the present hospital buildings were erected on the Thames Embankment, accommodation was provided for 75 nurses and 26 probationers, a total number of 101. During the present year the total of 175 has been reached, and it is with difficulty, and by all sorts of expedients, that they are accommodated. But provision is urgently needed for 28 sisters, 145 nurses, and 70 probationers, in all 243; and therefore it is felt that action cannot any longer be deferred. We have no doubt that the appeal in contemplation will show the public, in the most convincing manner, the necessity for the work.

### QUEEN'S NURSES AND THE ROYAL NATIONAL PENSION FUND.

We learn that the Council of the Liverpool Queen Victoria District Nursing Association have recently had their attention drawn to the fact that very few of the nurses engaged in attending to the sick poor in their own houses have sufficient, if any, provision made for their own support in old age, and that therefore they are anxious to assist and encourage the nurses in subscribing to the Royal National Pension Fund. With this object they have framed a scheme involving an expenditure of £4,000, which will secure a nurse at the age of 55 a small pension. The council hope hereafter to increase the amount, but they are to be congratulated upon making a beginning in the right direction. The assistance they propose to give is the more admirable because it will have the effect of helping nurses to help themselves.

### THE COMING CHRISTMAS.

Inquiries at University College Hospital, as to whether Christmas had yet been thought of, elicited from the matron the statement that it would not be thought of until about the 18th of the month. At the London Temperance Hospital, the new matron told our representative that she is collecting funds for the Christmas treat, but that she has not yet nearly enough money. She wishes the patients to have exactly the same pleasures as they had always enjoyed while Miss Lucas was matron, and she says that the difficulties of following such a lead as Miss Lucas gave are very great. Everything will be done as usual at the Middlesex Hospital to make Christmas a happy time for the patients; there will be good Old English cheer, Christmas trees, and plenty of flowers and plants in the wards, though the walls will not be decorated. The only difference will be that the patients will not be able to have their friends to see them, as visitors are not being admitted on account of the epidemic of small-pox in London.

### THE SHEPPEY GUARDIANS AND THE CHARGE NURSE.

There has been friction between the Isle of Sheppey Board of Guardians and the charge nurse at the Workhouse Infirmary for a long time, and we are glad to learn that at a meeting of the Board last week, Mr. Davy, one of the Local Government Board Inspectors, attended and expressed the opinion that "it must come to an end one way or the other." He proceeded to affirm "that for the proper discipline and management of the hospital Nurse Clark ought to have supreme command over her own department, and that her reasonable orders ought to be carried out by the ward attendant. The Sheppey Workhouse was in a most isolated position, and as it had always been found difficult in the past to get a good nurse it behoved the guardians to exercise every care in the matter or they might land themselves into the difficulty of being called upon by the department to engage a superintendent nurse. He thought that the medical officer ought to have power to say what, or what should not be done in the infirmary without appeal to the Board. If that were refused and he were in the medical officer's place he should resign the position." The only contribution of the Board towards the retention of harmony appears to have been the adoption of a motion that printed notices be put up in the infirmary stating the duties and hours of each person employed therein. We are not surprised that this course has not satisfied the charge nurse, and we hope that the authorities at Whitehall, acting upon the suggestion of Inspector Davy, will intervene.

### A TORONTO TRAINING SCHOOL.

On Friday evening, November 17, the graduating exercises of the Nurses' Training School connected with the Hospital for Sick Children, College Street, Toronto, were held. The training school is not large, because of the limited accommodation, but it is hoped that within the next two years a Nurses' Home will be built. The accommodation for the sick children has to be restricted to 195 beds, as two of the large wards are now used for nurses, in addition to 20 rooms on the top floor. In connection with the hospital is the Lakeside Home for Little Children of Toronto, which has accommodation for 150 children. A large number of friends were present at the exercises, and Mr. J. Ross Robertson, the Chairman of the Board of Trustees, presided. In the course of his speech he said that the training school has been in existence for very many years, but it was only in 1892 that it assumed its present form under the supervision of a trained superintendent. A hundred nurses had been trained during the past ten years, of whom about a score were superintendents and head nurses in Canadian and American hospitals. He congratulated Miss Louise C. Brent, the lady superintendent, upon the manner in which her staff discharged their duties. Dr. George A. Bingham enlarged upon the excellent quality of its graduates, and set forth the qualifications of a good nurse, namely—Health, intelligence, observation, connected with reason; tact, sympathy and cheerfulness; delicacy and gentleness; perseverance.

### UNIFORMS FOR USE.

It is just as well that the Local Government Board should have asked the poorhouse committee of Dundee Parish Council to make the rule of the house as to the supply and use of uniforms furnished to nurses clear to them at the time of engagement. As, however, we anticipated, the committee held that the entire uniform equipment issued to the poorhouse officials belongs to the Council irrespective of the period it may be in use. They have intimated their views to the Local Government Board, and in future there will be no occasion for any misunderstanding on the point, so far as the staff at Dundee is concerned.

### GLASGOW SICK POOR NURSING ASSOCIATION.

Last week we quoted some figures which show how highly the West of Scotland Co-operation of Trained Nurses is regarded in Glasgow, and the twenty-sixth annual report of the Glasgow Sick Poor Nursing Association, which was adopted at the meeting the other day, justifies the conclusion that the work of this organisation is not less appreciated. The staff now consists of 27 district nurses, 26 private nurses, and 18 probationers in training in hospitals, as well as three superintendents. In the year ending October 31st the number of cases treated by the district nurses was 2,641, and by the private nurses, 249. The income of the Association was £2,896 10s. 10d., as compared with £2,593 6s. 9d. in the previous year; and the deficiency £614 17s. 8d., as against a deficiency of £811 18s. 9d. in 1899-1900. This is better, but £600 is a big balance on the wrong side. We assume that the private nursing work does not add to the burden, but rather tends to diminish it. At the same time a resolute effort should be made by the supporters of the organisation to place it on a sound financial footing. The combination of the two departments in one institution is always open to the objection that the public get confused in their idea of its aim, and imagine that it ought to support itself out of the fees paid to the nurses.

### HOSPITAL SATURDAY FUND NURSING DIVISION.

Some inquiries having been made respecting the ladies in nursing uniform who sat on the platform at the annual meeting of the Hospital Saturday Fund at the Mansion House, we may state that they represented the "Nursing Division" of the Fund. They give their services for ambulance work on public holidays in streets and open spaces, finding their own uniform and bearing all expenses. In fact, they correspond to the men of the St. John Ambulance Association, and they are required to pass the examinations of that Association in first aid and nursing, with a re-examination every year. Recently these ladies were on duty on Lord Mayor's Day, and on the occasion of the return of the Heir-Apparent and his wife from their tour. Miss Mann, the lady superintendent, informs us that she has two vacancies for ladies wishing to devote their spare time to this work. The number is limited to 12. Last year it appears that 94 cases were attended on public duty; 61 not on public duty; cases "privately nursed," 108. As none of these ladies are hospital-trained, it is however a mistake to describe them as "nursing sisters," or the cases as "privately nursed."

### MATERNITY NURSING IN TOKIO.

Here are some of the subjects dealt with at the examination of midwives in Tokio in October, which an English nurse who lately arrived in Japan was required to answer. The importance of disinfecting and the mode; the management of the umbilical cord and its efficacy; the diagnosis and management of rectroflexible pregnancy uterus; the causation, symptoms, and management of hæmorrhage at the latter end of pregnancy; the symptoms and management of the many waters, containing fœtus, during pregnancy; the causation and symptoms of asphyxia of infant. It appears that the cord is managed in Japan exactly as at home, but that a patient of our correspondent, who had seen that the practice of not tying the cord on either the infant's cr the mother's side was recommended in an American book, was strongly inclined to follow the advice of the writer. The doctor in charge of the case at Tokio would not, however, allow it, and our correspondent is curious to know if any of our readers have seen the cord cut after the pulsation has ceased.

### WORTHING ISOLATION HOSPITAL.

We are pleased to learn, if at a rather long interval, that, although the Worthing Town Council, as stated in our issue of November 9th, declined to act on the recommendation of the Medical Officer of Health and appoint a nurse at a salary of £60 a year for Swandean Isolation Hospital, on the ground that she might not always be wanted, the hospital is not without a matron. The local paper which reported the proceedings did not mention that a trained nurse has been in charge of the hospital for four years, and is still there. She has nursed in that period, we are informed, over 300 cases, and no fault has ever been found with her. It is a matter for regret that she has not been given the permanent assistance which the medical officer thinks she needs, but it is satisfactory to find that the Swandean Isolation Hospital is not left without a trained nurse, although a staff of one can never be sufficient.

### A VERBAL PARTNERSHIP.

In defending a claim preferred in the Chancery Division of the High Court by Miss Theodora Tempsett for an equal half share in the profits, goodwill, and assets of the West Riding Nurses' Co-operation Home at Rotherham, Miss Gertrude Hirst denied that any profit had been made, and said she told the plaintiff in conversation before she joined the home that if at any time a profit was shown, her intention was that it should be apportioned amongst all concerned, including the nurses themselves, and that otherwise the home would not be co-operative. This was certainly a sound attitude to take up, and probably had a good deal to do with the decision of Mr. Justice Byrne, who, after hearing the whole of the evidence, delivered judgment to the effect that Miss Tempsett had failed to establish her case. But why did not Miss Tempsett, who avers that her nursing experience was to be set against the capital of Miss Hirst, have a written agreement? A "verbal partnership," the only kind alleged to have been concluded, is clearly absurd and unbusinesslike.

### THE SAN REMO INSTITUTE.

Miss Bryant, principal of the English Nurses' Institute and Medical Home, San Remo, held a reception the other day at her new villa, Sunny Bank, Via Borgo Pescio. Most of the leading medical men were present, and expressed themselves as being greatly pleased with the arrangements made for the comfort of indoor patients. The staff of trained nurses has been largely increased this winter, the new institution being much larger and more commodious than the old one in the Via Roma. It is an obvious advantage to English residents and visitors to be able to obtain at their own homes, when required, the services of trained nurses speaking in their own language. The open-air cure has, we understand, been specially arranged for, and can be carried out in every detail.

### WARRINGTON GUARDIANS AND THE NURSING ASSOCIATION.

The Warrington Board of Guardians have had under discussion the question of increasing their subscriptions to the District Nursing Association. The request was made on the ground that many cases of sickness which would otherwise come under the care of the guardians were attended to by the Association, the Board being to that extent saved the cost. Several of the guardians for outlying townships of the union contended that as the Nursing Association did not benefit their townships, they should not be called upon to subscribe to the Association, and an amendment was proposed that the subscription be not increased. The chairman explained that they were not in any way departing from their proper procedure in supporting an association of this kind, and we are glad to learn that, after some further discussion, the amendment was lost, the Board deciding to double their subscription.

### SHORT ITEMS.

The West Ham Council have tendered a vote of thanks to the superintendent of the Maternity Charity and District Nurses' Home at Plaistow, and her staff, for the services rendered by them in the borough in connection with the recent outbreak of enteric fever.—Amy, Lady Tate has made a gift of £1,000 in memory of her late husband, Sir Henry Tate, to the Queen Victoria's Jubilee Institute for Nurses, to be added to the donation given by Sir Henry, which is used for the benefit of the nurses themselves in times of sickness.

### Lectures to Nurses on Medicine and Medical Nursing.

By Norman Dalton, M.D. London, F.R.C.P., Physician to King's College Hospital.

### XXV.—CONVULSIONS (*continued*).—JACKSONIAN EPILEPSY—PARALYSIS.

In every case of convulsions the doctor has to discover the cause which is producing them. Nurses must, therefore, note particularly all that occurs in the fit. In the last lecture I pointed out the phenomena which occur in a true epileptic fit and in a hystero-epileptic fit; and these phenomena, if correctly observed and reported by a nurse, will enable a doctor to diagnose a case as one of epilepsy or of hystero-epilepsy. It is always particularly important to note whether the convulsive movements affect both sides of the face, both arms and both legs, or whether they affect one side only. In epilepsy and hystero-epilepsy both sides are convulsed. In the fits caused by poisons in the blood, such as uræmia, and those caused by reflex action as in teething, the fits also occur on both sides. They resemble the epileptic rather than the hystero-epileptic fits, because the movements are irregular and not at all "purposeful." But the fit does not pass through the regular stages of a true epileptic fit. Also, although both sides of the face and limbs are affected, they may not be convulsed at the same time; for instance, one side of the face may twitch, and then, some time after, the other may do the same. Or one hand may jerk and then the other, or the eyes may be turned to one side and then to the other. Lastly, in fits due to poisoning, the pupils, whether contracted or dilated, are practically always equal. It cannot be too often repeated that in every case of convulsions (or of recent paralysis) a nurse must always note the condition of the pupils. On the other hand, when convulsions are due to a "lesion" in the brain, they very often occur on one side of the face, one arm and one leg only, because such lesions as tumours or abscesses necessarily lie in one or other of the central hemispheres. Consequently only one half of the brain is irritated, so that only the side of the face, limbs, etc., which get their stimulus from that side of the brain exhibit convulsive movements. It must be remembered that the motor nerve fibres which pass from the brain to the spinal cord and so to the muscles, cross each other in the medulla. Hence the fibres from the right side of the brain go to the left side of the face and the left limbs, and *vice versâ*; and, for the same reason, a tumour which is irritating the right side of the brain will cause convulsions in the left side of the face, left arm, and left leg, and *vice versâ*.

The next point to note is that convulsive movements may not merely be one-sided, but that they may be limited to one part of one side. For instance, one side of the face alone may be affected, or one arm or one leg, or the face and the arm (on the same side) may be affected together, but not the leg, and so on. Now it is essential to note these limited movements accurately, because we have discovered that the muscles of the face, those of the arm and leg (and other groups of muscles, also) have each a centre on the surface of the brain; that is, there is an area in the brain from which come the impulses which in health set up the movements of the face, arm, etc. In disease, if the centre of the arm be irritated, convulsive movements occur in the arm; whereas if it be destroyed the arm becomes paralysed; and the same rule applies to the face and the leg. Consequently, if a nurse report convulsive movements of the right arm, we know that there is irritation going on at a particular spot in the left hemisphere of the brain; and if we think that the irritation be due to an abscess or a tumour, we can trephine the skull over the exact spot and open the abscess or remove the tumour. The above described limited convulsions are called Jacksonian epilepsy, after Dr. Hughlings Jackson, who detected their significance. The sequence of convulsions and paralysis is an especially important thing to note as it indicates an extension of the disease. For instance, if a tumour starts growing in the arm centre, it will cause convulsions in the arm. As it grows it will destroy the arm centre, and the arm will become paralysed. But, by this time, it will be encroaching on the face or leg centres; possibly on both, and then convulsive movements in these parts will be associated with paralysis of the arm. It may be as well to add that whereas the position of the movements tells us what part of the brain is being irritated, it does not tell us what the nature of the irritation is. This we have to learn by other symptoms: for instance headache, vomiting, and optic neuritis indicate that the cause of the irritation is a tumour. The same symptoms occur in cerebral abscess, but this also may give rise to remittent fever, and is usually met with in old-standing ear disease or in pyæmia.

Loss of the "motor functions" of the brain causes paralysis of the muscles, but paralysis may also be caused by disease of the motor paths in the spinal cord or of the motor nerves themselves. The word "hemiplegia" means paralysis of one half of the body and it only occurs in brain lesions, because it is only in the brain that the motor centres and paths which belong to one side of the body are so widely separated from those which belong to the other side (each occupying its own hemisphere) that a lesion on one side does not affect the other. In the spinal cord the motor paths of the two sides are so close to each other that quite a small lesion must almost necessarily affect both sides. Consequently, in most cases of spinal paralysis both legs are paralysed and the condition is called "paraplegia." In typical hemiplegia, the whole of one half of the body is not paralysed, but only the muscles of the cheek, lips and tongue, and those of the arm and leg. The muscles of the eyelids, the eyeballs, the trunk and many other parts escape paralysis. However, in certain cases they may be affected; for instance, if the lesion be in the crus cerebri, the muscles of the eyeball are paralysed on one side, and the facial muscles, arm and leg on the other. Hemiplegia most often results from hæmorrhage into the brain, or from plugging of an important artery in the brain by an embolus or by coagulation of the blood in it (thrombosis). The paralysed parts do not lose their power of feeling except in rare cases, and then, as a rule, only temporarily. The bladder and rectum are not affected in hemiplegia, provided that the patient is conscious.

Paraplegia is usually due to softening of the spinal cord (myelitis) or to crushing of the cord by the vertebræ which have become dislocated by violence or as a result of caries. Usually both lower extremities are completely paralysed, but if the disease destroy the higher parts of the cord, the whole trunk, the arms, and finally the diaphragm may be affected. Sensation is lost in the paralysed parts, because the sensory paths in the cord are destroyed as well as the motor, and no sensations can reach the brain. Also the rectum and bladder do not act properly. In some cases the bladder cannot contract at all, and even if a catheter be passed into it the urine flows feebly and it may be necessary to compress the bladder in order to empty it. In other cases it can contract, but does so quite independently of the will and without the patient being aware of its action. This difference in the mode of action of the bladder is important, as it helps the physician to locate the position of the lesion in the cord. In many cases of paraplegia, the paralysed limbs become rigid.

### Royal British Nurses' Association.

ANNUAL CONVERSAZIONE.

A large number of nurses and friends were present at the Kensington Town Hall on Tuesday evening, on the occasion of the fourteenth annual conversazione of the Royal British Nurses' Association. An excellent musical programme had been prepared, and an amusing farce was given by Miss Annie Esmond (Prince of Wales's Theatre), and Mr. Frank Lacey.

The guests were received by the Vice-Chairman of the Executive Committee: Miss Thorold, Sir James Crichton-Browne, Mr. Pickering Pick, and the Honorary Officers of the Association, Mrs. Coster, Mr. Langton, and Mr. Fardon. Among the company present were the following:—Miss Davies, Essex and Colchester Hospital; Miss Jones (Matron), General Hospital, Birmingham; Miss Pinchard (Matron), Children's Hospital, Paddington Green; Miss K. Scott, Sussex County Hospital, Brighton; Mrs. Turner, Lady Consul, Rochdale; Miss Squire, Women's Hospital, Soho; Mrs. Price (Matron), Consumption Hospital, Brompton; Miss Hannaford, Poplar and Stepney Sick Asylum; Miss Jones, Chest Hospital, Victoria Park; Miss Peter, Queen Victoria Jubilee Institute; Miss Hamilton, University College Hospital; and Miss Spencer, Alexandra Hospital for Children. Several nurses who have recently been in South Africa were conspicuous by their medals, and one army scarlet cape was noticeable among the more sombre blue and grey uniforms; this was worn by a sister who returned from South Africa about six weeks ago. There were a few gentlemen present.

The programme was evidently very much enjoyed; it opened with a selection by the Æolian Ladies' Orchestra, under the conductorship of Miss Rosabel Watson, Miss A. V. Mukle accompanying on the piano. Miss Mina McDonnell and Miss Helen McCarthy gave a vocal duet. The former being a soprano and the latter a contralto, the voices blended very pleasantly; both ladies afterwards sang alone, and were warmly applauded, and so also was Miss Louie Heath in her pianoforte solos. Mr. Norman Ridley and Mr. Stepney Rawson gave some effective songs; the former obtaining great applause by the slow and sustained "Drink to me only," followed by the quick and lively "Quand ero paggio," and the latter in the contrasted old English songs "Why so Pale and Wan?" and "The Pretty Creature." The one-act farce "He and She" was very much appreciated; "She" did all the talking, while the unhappy "He" tried vainly to get in a word edgeways, in explanation of his absence for the whole of the anniversary of their wedding day, until the brilliant idea of showing his wife the present he had been to buy occurred to him, after which there was a reconciliation. The clever acting of the tongue-tied husband elicited laughter. Mr. Lacey afterwards gave a humorous recitation. A vocal quartet by the two lady and two gentlemen singers brought a very pleasant evening to a close.

NURSES AND THE OPEN-AIR TREATMENT.

Miss Jane Walker, M.D., lectured to a large number of nurses on Monday afternoon, at 10 Orchard Street, on the "Open-Air Treatment of Consumption." The chair was taken by Dr. Bezly Thorne, who introduced Dr. Walker as the pioneer of the open-air system in this country.

Speaking on the actual nursing of consumption, after a historical sketch of theories and methods, Miss Walker observed that doctors and nurses very rarely contracted the disease from a patient; and, it was not too much to say that, providing the room occupied by the patient was kept clean and open, and all waste animal matter at once destroyed, so far as present knowledge went, there was no risk whatever to another person sharing the room. The danger was, however, very great in confined spaces. The open-air system was the system of common sense; it meant abundance of nourishing food, fresh air, exercise, and freedom from worry and anxiety. Fresh air was not so much a difficulty as the requisite amount of food, for consumptives required more than ordinary mortals. At the sanatoria with which she had to do in Suffolk, the servants had instructions not to remove the plates until they were cleared. The nurse with excellent hospital training sometimes failed signally in persuading the patient to eat, while another with inferior credentials might succeed.

At the close of the lecture, which was listened to most attentively, and warmly applauded, several questions were asked. One nurse wanted to know whether massage would be helpful in cases where such large quantities of food had to be taken; another asked whether consumption was hereditary; Miss Leigh inquired whether excessive open-air brought on rheumatism; and Mrs. Dacre Craven asked how a nurse already trained was to get special training in nursing consumption; how, for example, could she learn to persuade her patients to eat. Mrs. Craven said that it was a new thing to her to hear that consumptives ought to eat more than they wanted, and she would like to know how it was done.

Dr. Walker, in replying, said she did not think that any nurse who had not had sanatorium training knew what was meant by open windows; they were so desperately afraid their patients would catch cold. With regard to persuading the patients to eat, it seemed to be largely a matter of tact. As to whether the disease was hereditary, it was a difficult question, but it was safe to say that when it was known to have occurred, the other members of the family should exercise the greatest possible care. Massage had not been found particularly beneficial, especially as consumptives required absolute rest. Consumptive patients did not suffer from rheumatism, and they soon got used to the open windows.

The meeting terminated with the usual vote of thanks.

### Death in Our Ranks.

We record with regret the death of Nurse Emma Hollis, of the Nurses' Home, 50 Bethel Street, Norwich, of typhoid fever, contracted in the discharge of her duties. "Death," a correspondent who knew the deceased writes, "has re-removed a nurse whose devotion to duty, kindness to all around her, and gentle influence in the home endeared her to all, and her presence will long be missed." Miss Hollis received her training at Miss Davidson's Home for the Dying, Victoria Chest Hospital, and St. Mary's Hospital, Paddington, and afterwards served as private nurse on the Norfolk and Norwich Staff of Nurses eight years. She was buried at Leicester, where her family reside, on December 2nd.

### THE NURSES' BOOKSHELF.

Lessons on Massage. By Margaret D. Palmer. (London: Baillière, Tindall and Cox.)

We regret that in a review of this book which appeared on November 30th we inadvertently stated that the treatment of fractures by massage was not alluded to. We find, however, on pages 195–196, that an account is given of this method, describing the necessary precautions, and the manipulations required. Perhaps we may say by way of explanation that, notwithstanding the somewhat full index with which the book is provided, the word "Fracture" does not appear in it.

### The Nurses' Co-operation.

### PRESENTATION TO MISS HUGHES.

The Winter Garden at the Portland Hotel was, on Wednesday afternoon, the scene of the presentation, to Miss Amy Hughes, of the diamond ring and gold watch which we described in last week's issue. Red-shaded lamps and palms, with easy chairs and oriental tables, made a picturesque setting for the nurses of the Co-operation, who mustered some 150 strong to show their loyalty to their late superintendent.

Shortly after three o'clock, Miss H. E. Roberts, standing on the steps so as to be visible to all present, read the following speech:—

"Miss Hughes, we have asked you to meet us here this afternoon that we might present you with a diamond ring and a gold watch as a small token of our affection and esteem for you. During the four years in which we have enjoyed the privilege of your wise and kindly rule as superintendent of the Nurses' Co-operation, we have always felt that in you we had a friend who was ready to help us in our difficulties, and to sympathise with us both in our troubles and pleasures; a just friend who would unhesitatingly point out our faults to us, or give us a word of encouragement when we needed it. We should like to take this opportunity of thanking you for all you have been to us, and we can only add how deeply grieved we are that you have thought best to leave us, and how sincerely we all wish you health, prosperity, and every happiness in the future."

Miss Roberts then presented the watch and ring; and Miss Hughes was heard to say, "I must look at them, please, before I say 'thank you.'" She replied as follows:—

"My Dear Friends,—I hardly know how to thank you for the kindly feeling expressed in the speech just read. I am deeply touched by the affectionate appreciation of the services I was able to render you while Superintendent of the Nurses' Co-operation. I thank each one of you most warmly for these beautiful presents, so valuable in themselves, and doubly so because of the feeling which prompted the gift. It only makes me feel how much I left undone and how little I realised the importance of the necessarily brief interviews I have had during the past four years with the many nurses of this great Association. It was not easy always to meet each of you on your own ground, and how often I failed you and I both know. I am only thankful for the way in which you have taken the will for the deed, and also deeply grateful for the patience and kindly spirit with which you accepted my attempts at advice and admonition.

"The number of nurses belonging to the Co-operation constitutes at once its strength and its weakness. Its many members contribute to its widespread renown and large area of work, and yet at the same time this feature opens the door to many difficulties.

"I have learned much while with you. It did not take four years to find out the peculiar temptations and needs of your daily lives, and also the causes which lead to your being so often misunderstood. It makes one's anger wax hot to hear the trivial complaints, the impossible ideas, about the work and position of the 'trained nurse' when one knows what devoted, self-sacrificing, unselfish lives so many of you are leading. I honestly believe the private nurse has the most trying and the most thankless part of the profession, taking her work on the whole. At the same time in your own hands lie the remedies for many of the troubles—all the difficulties come into focus sooner or later at the office, and generally cause and effect fall there into their relative positions, and one can see where the mistake has been. I would like to bear testimony here to the absolutely just way in which complaints are there investigated and considered. Nothing is done hurriedly, every point for and against is duly weighed, and no final decision is arrived at until the nurse has been given ample opportunity for explanation. The whole process is fair to the nurse, her employers, and the Co-operation as a whole. I think American nurses have solved one of our problems in the special training given in hospitals for private nursing. Nearly every hospital has wards for paying patients, generally numerous well-appointed single bedrooms, where the patients are nursed under the same conditions as at home. Their friends are allowed free access to them, their food is specially prepared and daintily served, every detail of a toilet more or less elaborate is attended to by the nurses as part of the two or three years' training. Such nurses, when they begin work on their own account, are naturally more adaptable than those who have to buy their experience. So far as I was able to ascertain co-operation in our sense of the word does not exist among private nurses. Nearly every training school has now its Alumnæ Association composed of the graduates, or, as we say, trained nurses from the hospital, who join as soon as the training is over. The nurses are admitted under certain conditions, and pay an annual fee. They meet to discuss nursing questions, in many associations have a benevolent fund (in one case an endowed private bed in the hospital for any sick associate), and generally unite to further the interests of the nursing profession. In connection with these associations registries of its members are opened at their own hospitals, the nurses paying a fixed annual fee for working expenses. The rules I saw were almost identical with those of the Co-operation as regards being ready for work when booked, not refusing cases, etc. As the nurses have all been in the hospital, and are known to the matron and the medical staff, there is less difficulty about special cases than in our larger societies, where nurses join from various training schools with different methods and traditions. Thus to a certain extent the American private nurse has her path smoothed for her, and we congratulate our transatlantic sisters it is so. But we need not think ourselves handicapped in the race. The very fact we have to assume more personal responsibility for our work develops at once the best and the worst side of each nurse's character. This is what I meant by saying you hold so much in your own hands.

"Will you think it too candid if I say I think half your troubles arise because you fail to put yourselves sufficiently in the place of the patient and even more in the place of the friends? It is a great demand to make, a heavier one than many would believe possible without realising the constant strain imposed by the fact of always going to new people and new surroundings. I know what a nightmare the setting-out for a new case is to some, even after the experience of years; but, believe me, this is the key to the position. No nurse who enters a household in that spirit can be accused of hardness, selfishness, want of consideration, nor considered a 'necessary evil' to be displeased with at the earliest opportunity. It is not possible for every nurse to be acceptable in all circumstances; quite involuntarily she may prove a 'square peg in a round hole,' but there is a great difference between this and 'antagonising,' as the Americans say. It is not the professional skill alone, not the disclipine of training, but the thoughtful womanliness over and above this which will remove the cloud that rests on the title of 'trained nurse.' I may never have the opportunity of speaking to you again, and I would urge each one of you to take hold of this idea of individual responsibility more clearly and more strongly than ever before. You owe it to yourselves, to your self-respect, in the guarding of yourselves from even a suspicion of light or foolish conduct, harmless enough in a private position, but bringing disrepute upon your profession. You need it in the guarding of your health, which is really your capital, especially in taking your due amount of sleep, not curtailing the time to go out with friends, etc. It is the lassitude and irritability due to want of sleep that brings other evils in its train—drugs and stimulants to whip up the jaded mind and body—and once that habit begins a nurse is fast drifting out beyond help.

Of your responsibility to your patients I need not say much. Your training helps you there; but let the motive of the responsibility be a higher one than a mere barter of your skill and time for its monetary value. Let every patient be a human being, not a *case*, given into your charge for the time to do your level best for, and as you would be done by.

Then your responsibility to the Co-operation is a grave one, and you should each accept it in its widest sense. By its constitution every one of you has an individual interest in it, in its work and in its management, and each of you represents it to the medical profession and the general public. On each of you rests the reputation of the nurses as a whole, for every family you enter judges, and naturally so, of the whole body by you and the impression you give. It is a grave but a grand responsibility, and knowing you all as I do I am not afraid to trust the future of the Co-operation in your hands, and through it the interests of the nursing profession as a whole. You are a representative body of trained nurses, drawn from every part of Great Britain, and though much is expected of you, you can more than fulfil the expectation if you will. Thank you for listening to me so patiently. I can only say in conclusion what an honour it was to leave my post with you, and to ask you to believe the step would not have been taken had it been possible for me to do otherwise. Sure of your affection and interest in my future, I may tell you I am shortly to begin work again, but I trust never to lose my interest in the Co-operation, nor lose sight of the many friends whose kindly sympathy helped me so much in the responsibilities of that position. Once more I thank you all.

The speech, which was listened to most attentively throughout, was punctuated by the nurses' remarks here and there. "Quite true," was heard in an aside, when a cloud was spoken of as hanging over the title of "trained nurse;" and there was much laughter at more of Miss Hughes' remark, in parenthesis, "The *hers* among patients are more difficult than the *hes*."

And when, as a finish-up, Miss Hughes declined to say "Good-bye," because she could not, the nurses applauded warmly, and one or two remarked, "It does seem hard, doesn't it?"

After the presentation, the meeting resolved itself into a social gathering, and tea and coffee were handed round. The proceedings throughout were enthusiastic; and both before and after the event of the afternoon, everyone was anxious to shake hands with Miss Hughes, who greeted each nurse individually in the warmest possible manner.

### Our Christmas Number.

OUR Christmas Number will be issued next week, December 14th, and will consist of a series of sketches, written by nurses, of Christmas in Hospitals beyond the Seas. In addition to the letter-press there will be several half-page illustrations. Orders should be given at once to the publishers.

### Appointments.

ASHTON-UNDER-LYNE INFECTIOUS HOSPITAL. — Miss Baxter has been appointed matron. She was trained at the Bolton Borough Fever Hospital, and has since been assistant nurse at Stockport Isolation Hospital, nurse at Birkenhead, charge nurse at the Sunderland Sanatorium, superintendent nurse at Houghall Fever Hospital near Durham, and for the last 18 months matron at Hebburn-on-Tyne Fever Hospital.

EAST PRESTON UNION INFIRMARY, NEAR WORTHING.—Miss Sarah Holloway has been appointed nurse. She was trained at Oldham Infirmary, and has since been nurse at the Brentford Union Infirmary.

FEVER HOSPITAL, BLACKBURN.—Miss Lizzie Thomson has been appointed charge nurse. She was trained at Middle Ward Hospital, Motherwell, and has since been charge nurse in the same institution.

GOOLE COTTAGE HOSPITAL.—Miss Mary S. Webster has been appointed matron. She was trained at North Devon Infirmary, Barnstaple, and has since been nurse successively at the Cottage Hospital, Chorley; Macclesfield General Infirmary; the North Devon Infirmary, Barnstaple; and Bridgwater Infirmary.

IVER, LANGLEY, AND DENHAM COTTAGE HOSPITAL.—Miss Blanche M. Parker has been appointed nurse-matron. She was trained at the London Hospital, and has since been charge nurse at the Hospital St. Cross, Rugby, sister at the Royal Infirmary, Bristol, and assistant matron at Leicester Infirmary.

LLOYD COTTAGE HOSPITAL, BRIDLINGTON.—Miss M. Cecil Lewis has been appointed matron. She was trained at the Royal Albert Edward Infirmary, Wigan, after which she was appointed sister of women's wards, and has since, for two years, held the post of senior sister and deputy matron at the Southport Infirmary. Miss Lewis holds the L.O.S. certificate, and has had experience in private nursing.

PORTSMOUTH WORKHOUSE INFIRMARY. — Miss Mary Hartley and Miss Emily M. Robson have been appointed charge nurses. Miss Hartley was trained at Bradford Union Infirmary, and Miss Robson at Birmingham Infirmary.

ROYAL ALBERT EDWARD INFIRMARY AND DISPENSARY, WIGAN.—Miss Louise François de Chaumont has been appointed sister of women's wards. She was trained at the Royal Albert Edward Infirmary, Wigan, from 1891 to 1894, and previous to her present appointment she worked for five years in connection with the Bradford Eye and Ear Hospital, first as assistant nurse and subsequently as charge nurse. Miss Warburton has been appointed sister, not nurse, in the same institution.

WANDSWORTH AND CLAPHAM INIFRMARY.—Miss M. Elliott has been appointed home sister. She was trained at the Western Hospital, Glasgow, was subsequently charge nurse at Lady Hope's Convalescent Home, Lanark, and has since been ward sister and night superintendent at Southwark Infirmary, East Dulwich Grove.

WEST BROMWICH ISOLATION HOSPITAL.—Miss Gertrude Poole has been appointed charge nurse. She was trained at the City Hospital, Lodge Road, Birmingham, and has since been nurse at the South-Eastern Hospital of the Metropolitan Asylums Board. She has also done holiday duty at the District Hospital, West Bromwich.

WEST DERBY UNION INFIRMARY, LIVERPOOL. — Miss Laura M. W. Clayson has been appointed home sister. She was trained at the Royal Devon and Exeter Hospital, where he has since been staff nurse and operating sister.

WINCHESTER UNION INFIRMARY.—Miss Jane Crane has been appointed superintendent-nurse. She was trained at Southwark Infirmary, East Dulwich, and has since been nurse at St. Pancras School Infirmary, Leavesden, and charge nurse at St. Pancras Infirmary.

### American Women and the Buffalo Delegates.

The Society of American Women in London, represented in particular by Mrs. Dietz Glynes, the acting president, and Mrs. Arthur Fay, the vice-president, entertained the British delegates to the International Congress of Nurses at Buffalo at the rooms of the Society at "Prince's," Piccadilly, on Friday afternoon last. Amongst the delegates present were Mrs. Bedford Fenwick, hon. president of the International Council of Nurses; Miss Isla Stewart, matron of St. Bartholomew's Hospital; Miss Amy Hughes, late lady superintendent of the Nurses' Co-operation; Miss McGahey, matron of the Prince Alfred Hospital, Sydney, representing the Australasian Trained Nurses' Association; Miss Cartwright, of the Registered Nurses' Society; Miss C. J. Wood, and Miss Waind, of the League of St. Bartholomew's Nurses. The hall was well filled, about half a dozen nurses in uniform being noticeable among the audience.

Mrs. Glynes, having expressed the pleasure of the society at being able to welcome the delegates, read a letter from her Royal Highness Princess Louise, thanking the society for the invitation they had sent her, and regretting that absence from town obliged her to decline it. The Princess wrote that "she had always been greatly interested in nursing, and would have much enjoyed being present had it been possible." Letters of regret were also read from Lord George Hamilton and Mrs. Joseph Chamberlain.

Mrs. Bedford Fenwick, was the first speaker. Her subject was "The benefit of international co-operation amongst nurses, and what it is going to do for nurses in the future," and she traced the gradual growth of the international idea, which, she said, first suggested itself to her in 1892 when she was visiting America. Seven years later, at the International Meeting of Women in London, the idea sprang upwards and bore fruit, and the meeting of nurses at Buffalo was arranged. Mrs. Fenwick urged the desirability of some uniform basis of education and standard of training for nurses, and also that, if possible, there should be some preparatory training before going into the wards, as well as opportunities for attending post-graduate lectures after qualification in order to keep the information acquired up to date. She thought that one of the most interesting features of the Buffalo Congress was the comparison of the different systems employed by Great Britain and America respectively in nursing their soldiers and their sailors, and she inclined to the belief that the scheme organised by England gave more scope for efficient organisation than that of the United States. In conclusion she alluded to the kindness she had received in America, so that now upon her return she felt as if "her dear Transatlantic cousins had dusted and polished her brain and sent it home as good as new."

Miss Isla Stewart, who described nursing as no longer simply an occupation for the display of self-sacrifice, but "as an honourable calling for honourable women," gave some details of the John Hopkins Hospital at Baltimore, whose executive were acting as pioneers in the giving of preparatory instruction to nurses before entering the wards. Mrs. Fenwick had touched upon the same subject in her address, though she admitted that the preliminary training was not likely to be universally adopted because of the expense involved. Miss Stewart cited a long list of the subjects taught to the 16 probationers who enter for preliminary training. The first six weeks they act as housemaids and have certain bedrooms put under their charge for which they are entirely responsible, both for the rooms themselves and for the linen used in them. The next six weeks they train in the dining-rooms learning parlour work, followed by three months in the kitchen. Here they begin by doing kitchenmaids' work, cleaning the grates and flues, seeing to the sculleries and the sinks, the pots and the pans, and afterwards not only learn to prepare and cook the meals for the nurses and the invalids, but how to purchase the food, and to keep and turn it to the best advantage when bought. In addition to this the students gain a knowledge of sanitary work, plumbing, ventilation, heating and lighting. Thus fully instructed in domestic matters they pass on into the wards where they remain for two and a half years more. They have no salary but are given uniform, books and stationery, and can win money scholarships at the examination. They work for eight hours a day in the wards, the rest of the day being devoted to rest, recreation, and study.

Miss Catharine Wood spoke on the nursing of the insane. Whilst she was in Buffalo she saw all over the Buffalo State Hospital for the Insane, and came to the conclusion that matters in America are in much the same transition stage as in England, viz., that the attendants employed to tend the sick in mind are no longer of the low uneducated type of former days, but neither are they specially trained for their work. In the infirmary attached to the asylum the nurses are given a certain amount of training, but of a very circumscribed character, and in no way equal to that obtainable in a general hospital. The working of the Lunacy Laws is simplified in the United States, because there are only two bodies which have any voice in the matter—the Commissioners of Lunacy who have to see to all practical details, and the Treasury which provides the necessary funds. Three classes of patients are received in nearly all the asylums under the same roof: (1) the destitute; (2) those who can pay a little; and (3) those who can afford the full charges, which never exceed $10 a week.

Miss Amy Hughes alluded to district nursing in America, and said that during her stay she had seen as much as possible in the time. In New York the work is especially arduous, because of the enormous proportion of emigrants. The tenements she described as far worse than English slums, because the slums here are in a large measure the result of age, and are fast being cleared away, whereas the tenements are new, and with handsome frontages to the street have the most fearful backgrounds. The houses are enormously high, and are built round a shaft only six feet across. The bedrooms all open on to the shaft, which is the sole means of providing ventilation and light for thousands of men, women, and children even in the hottest weather. In these awful rooms the nurses have to tend the sick and dying. In Chicago matters are somewhat similar, though under modified conditions. The Victorian Order of Nurses in Canada, continued Miss Hughes, works most nearly on the lines of our Jubilee nurses, though their work amongst the settlers is a distinct feature. Often the nurses have to go 100 miles to attend a single case. To simplify the nursing as much as possible arrangements are sometimes made to receive patients at the home where the two nurses reside, which thus partakes somewhat of the character of a cottage hospital. In conclusion Miss Hughes remarked that she was much struck with the reverence in which the name of Miss Florence Nightingale is held in America; no less than in England it is a household word, and so glibly were Miss Nightingale's tenets quoted by many, that she herself had to look up references when she returned.

Miss McGahey made allusion to the fact that at the Prince Alfred Hospital, Sydney, the curriculum for the last—the fourth—year of the training of nurses includes housekeeping, dispensing, and midwifery.

The subsequent proceedings were of a social character.

### Everybody's Opinion.

[Correspondence on all subjects is invited, but we cannot in any way be responsible for the opinions expressed by our correspondents. No communication can be entertained if the name and address of the correspondent are not given as a guarantee of good faith, but not necessarily for publication. All correspondents should write on one side of the paper only.]

### THE NURSE'S NEVER.

"Nurse L." writes: There seems a good many willing to find fault with the "Nurse's Never," but my experience tells me that a great number of nurses want to be reminded of things they should "never" do. I, too, have had similar experiences to that of "G. M.," *re* putting hot-water bottles next the patient's skin minus any covering; and although it was not followed by any serious results, it was certainly not the correct thing to do. Then, again, I have seen a feeding-cup near the patient's bedside with milk or food of some kind therein and strong carbolic acid in the bed-pan when given to the patient; the temperature taken at midday when it should have been taken in the early morning; and very often a patient handled roughly by a trained nurse. Surely such nurses want someone to remind them of what they should never do. I sincerely hope when the medical lecturer has more time to spare. he will continue the "Nurse's Never" and take no notice of those who find fault or of those who are too old to be told.

"A Royal Alexandra Nurse" writes: I think the matron, writing in last week's Hospital, must have a very poor opinion of nurses in general. I do not think any nurse ought to want reminding of what she is supposed to have thoroughly learnt during her training. If so, why don't other people want reminding also? It would sound very silly to remind an engine-driver to be sure and look at his signals, or a cabdriver to look where he was going, or a postman to look well at his letters. And surely it is not to a matron's credit to admit that nurses do want reminding of their nursing rules. I think it would be most unfair of any matron to send a nurse to a patient who was paying for her services, feeling all the time she might perhaps burn the patient with a hot-water bottle, or give an over-dose of medicine, or make a drying-ground of her patient's bed-room, or some other such silly things. I am glad to say that our matron has a better opinion of her nurses. I belong to a nursing home where there is a large staff of nurses, and we were rather amused at the way we were hit at, and concluded that the author had never been ill and required the services of a trained nurse. Perhaps when he has required them, he will direct his "Nevers" to probationers.

### NECESSARY AND UNNECESSARY WARD WORK AS TRAINING FOR PROBATIONERS.

"Pennie" writes: In 1873 I entered the Highgate Infirmary as a Nightingale probationer. We found plenty to do in nursing the patients without doing any housework, even though as "Sophia" points out there was no sterilising to do. A ward assistant was attached to each ward, and convalescent patients were often pleased to help with the taps, etc. The only thing in the way of scrubbing we had to do was the mackintoshes. We breakfasted at 6.30, went on duty at 7, had two hours off in the afternoon, and off duty altogether at 8.30. What with waiting on the patients, attending two lectures and two classes a week, we were always glad when bed-time came, and often wondered how on earth pro's got on who had rough work to do as well. Since then I have nursed in cottages, at infectious cases, etc., and have found I could do whatever was needed, if there was no other person to do it, knowing that such cases only last a few weeks or months at the most, and that afterwards I should have rest in the home. I knew a farmer's daughter who would have made a splendid nurse; she went as a paying probationer at a large London hospital, and left at the end of three months because, as she remarked to M., "I can learn housework at home, so it is not worth while paying a guinea a week to learn."

### WHY ARE NIGHT NURSES NEGLECTED?

"E. C. Sandford" Matron and Superintendent of Nurses at the City Hospital, Edinburgh, writes: I am much pained at the tone of "Yorkshire's" letter. A great daal of what she says may be true, but a great deal is not, and it is a letter calculated to raise bad feeling between matron and nurses. The training nowadays may not be so hard, but the time is much longer, the competition so keen, that promotion is more difficult to obtain, and therefore nurses are subjected much longer to the somewhat school-like discipline of the rank and file than in the days of yore. And it is not only in hospital, but in all other conditions of life, that the craving after luxury exists. In the old days children only got jam once a week, now they have it every day; is it a wonder that nurses crave for their piece of jam? I do not think that "coarse" is a word to apply to our nurses of the present day.

"Another Matron" writes: I think "Yorkshire" is a little hard on the nurse of to-day, although I agree with her that the more refined type of nurse endures more and says less than she who has not had the advantages both of education and home life of her more fortunate sister. More is demanded of a nurse nowadays than was the case some ten years ago, and if more is wanted of her more care and attention must be given to her. I think she often has good reason to complain even if she does not do so, for food is sometimes badly cooked and badly served, although of good quality. I order all food for my nurses, and, further, I see that they get what I order, properly served and cooked, and they do not grumble. Perhaps I am unusually fortunate in my nurses, but I have been a matron for five and a half years (albeit of a small hospital) and I feel sure that on the whole they are contented. Of course, there are people who must grumble at something—if not food, time off duty, perhaps, is their grievance—but these people are not nurses nor will they ever be, so the sooner they are assured they have mistaken their vocation the better for patients and matrons alike.

"N. S." writes: Having had several years' experience of night duty, I feel that I should like to give my ideas with regard to night nurses' diet. I do not think that nurses are deteriorating quite so much as "Yorkshire" seems to think they are, and why they should be considered selfish because they sometimes express a wish for a little variety in their food I cannot imagine! It is certainly a subject which matrons might think a little more of. I have found nurses of the "refined" and "unrefined" type alike complain of having eggs and sardines alternately night after night, with an occasional slice of cold mutton to break the routine. We do not expect to live in hospital as most of us do at home, but it has always struck me that "anything" will do for the "night nurses." Perhaps "Yorkshire" has been fortunate enough to belong to a hospital where night-nurses are considered. There are a few I will admit, as I was in one myself, belonging to the Metropolitan Asylums Board, where the medical superintendent and committee used often to ask the night-nurses if they had all they wished for during the night, and the constant change in their diet was very much appreciated. As for remarks made by "Yorkshire" about future advertisements appearing with items such as "good chef kept," etc., inserted, well! one would not be likely to look to "her" to uphold the nursing profession!

"Ex-Nurse" writes: Having been trained in one of the largest general hospitals in London I can speak feelingly with regard to the night nurse. There is no need for me to reiterate what others have written, but in the matter of food I do not think that sufficient thought is given to the total change in one's surroundings as compared with day work. It is not so much a question of quantity or quality as of the conditions under which it has to be taken. My experience has been that one's appetite is much more capricious during the hours when most of us are accustomed to be asleep, and that then even the "sausages, herrings, and eggs" mentioned by a "Matron who was once a Night Nurse" would none of them be such a "special relish." It was always a wonder to me that arrangements could not have been made for a couple of the kitchen staff to take night duty by turn in order to prepare the nurse's meal in the dining-room in the regular way

(it need not be elaborate) so that those on duty could go in reliefs for a half-hour's meal. Again, there was the attendance at Chapel on Sunday morning made compulsory for the night nurses, so that going on night duty at half past eight on Saturday evening we did not get away from the hospital (our sleeping quarters being at the home) till 11 A.M Sunday morning. I am sure we never benefited one scrap by those services, for Dame Nature will have her way and the Chaplain must often have seen the nodding heads on our side of the Chapel. If the Chapel was so essential for night nurses, why could we not have been permitted to choose the evening service when we could come fresh from sleep and rest before taking on the next night's duty? I once mentioned this to the Chaplain but nothing came of it; I suppose he thought that it was altogether too trifling.

SCARCITY OF NURSES IN WORKHOUSES.

"A POOR LAW GUARDIAN" writes: Under the above heading in your issue of the 2nd inst. "A Workhouse Nurse" has delivered herself upon what she conceives the conduct of the workhouse—reading between the lines—more particularly relating to the infirmary. "A Workhouse Nurse" states she held the post of head nurse in a country union. A rather important omission is made in this statement, inasmuch as your readers are not made acquainted of the fact as to what length of term is to be here inferred. Then, too, of still more importance, "A Workhouse Nurse" might have stated the length of service which stood to her credit under the Poor Law system of this country. Sir, yet another point occurs—readers of your paper may have asked the question to themselves, Why did "A Workhouse Nurse" leave her situation? To me, the only explanation, reading her letter with an analytical mind, may be summed up in the one word dignity or status as applied to the opposing word "untrained," which weakens the trend of her effusion. Undoubtedly "A Workhouse Nurse" has in her mind the Local Government Board Order of 1897; it is unnecessary to quote the order, to which may be added the three years' requisite training. One of the misfortunes under which "untrained and uneducated matrons"—if such there be—suffer is that they were born a little too soon, *i.e.*, before the Education Act of 1870, being thus deprived of the benefits accruing from a School Board education. What shall be said of the three years' hospital training? To the intelligent nurse it should prove of immense service, to another merely a weapon for illegitimate agitation and so stultifying the advancement of "A Workhouse Nurse's" cause. "A Workhouse Nurse" in her letter pointedly puts the following question: "What can guardians expect if they allow untrained, uneducated matrons to interfere with trained nurses' duties." To this the Order of 1897 does not refer, for the matron and master's duties are as hitherto, the same power being allowed to them and in no sense weakened. It will justly be admitted that the majority of Poor Law Guardians are men of experience and profiting by experience, many of them having occupied their seats for twenty and even thirty years on their various boards, I feel sure there is little knowledge to be gained from your correspondent's letter. The Poor Law System in the management of both house and infirmary can only be dealt with satisfactorily by long years of experience, which I fear, from the tone of her letter, leaves "A Workhouse Nurse" a poor and inept pupil.

### To Nurses.

**WE invite contributions from any of our readers, and shall be glad to pay for "Notes on News from the Nursing World," or for articles describing nursing experiences, or dealing with any nursing question from an original point of view. The minimum payment for contributions is 5s., but we welcome interesting contributions of a column, or a page, in length. It may be added that notices of appointments, entertainments, presentations, and deaths are not paid for, but that we are always glad to receive them. All rejected manuscripts are returned in due course, and all payments for manuscripts used are made as early as possible after the beginning of each quarter.**

### For Reading to the Sick.

"SWEET PATIENCE, COME."

SWEET Patience, come:
Not from a low and earthly source—
Waiting, till things shall have their course—
Not as accepting present pain
In hope of some hereafter gain—
Not in a dull and sullen calm—
But as a breath of heavenly balm,
Bidding my weary heart submit
To bear whatever God sees fit.
Sweet Patience, come!

*Hymns of the Church Militant.*

Each cross has its incription, and on mine
Was written legibly, "That which I do
Thou know'st not now, thou shalt hereafter know."
And each cross has its well, deep, deep below,
And inexhaustible—a well of life
For after-days of drought and barrenness.
Each cross, too, has its rainbow-light on cloud
Cast sweetly down, that all may not be dark.

*Bonar, D.D.*

"Thou cam'st not to thy place by accident:
It is the very place God meant for thee."

There is no haphazard in this world. God leads every one of His children by the right way. He knows where and under what influences each particular life will ripen best. One tree grows best in the sheltered valley, another by the water's edge, another on the bleak mountain-top swept by storms. There is always adaptation in Nature. Every tree or plant is found in the locality where the conditions of its growth exist; and does God give more thought to trees and plants than to his own children? He places us amid the circumstances and experiences in which our life will grow and ripen the best. The peculiar discipline to which we are each subjected, is the discipline we severally need to bring out in us the beauties and graces of spiritual character. We are in the right school. We may think that we would ripen more quickly in a more easy and luxurious life, but God knows what is best; He makes no mistakes.

*J. R. Miller, D.D.*

Interpose no barrier to His mighty life-giving power, working in you all the good pleasure of His will. Yield yourself up utterly to His sweet control. Put your growing into His hands as completely as you have put all your other affairs. Suffer Him to manage it as He will. Do not concern yourself about it, nor even think of it. Trust Him absolutely and always. Accept each moment's dispensation as it comes to you from His dear hands, as being the needed sunshine or dew for that moment's growth.—*H. W. S.*

My life is but a weaving
Between my God and me,
I may but choose the colours
He worketh steadily.

Full oft He weaveth sorrows,
But I in foolish pride
Forget He sees the upper,
And I, the under side.—*Anon.*

### Echoes from the Outside World.

AN OPEN LETTER TO A HOSPITAL NURSE.

QUEEN ALEXANDRA'S birthday (December 1st) follows very soon after that of her Royal consort, but as it fell upon Sunday this year, the celebration was postponed till the following day. Many congratulatory telegrams were sent, one from the Lord Mayor of London, which the Queen graciously acknowledged, and there was a good display of illuminations in the evening, Pall Mall, in particular, being a blaze of light. At Marlborough House it was noticed that there was a fresh device, "A. R." picked out in tiny gas jets, with a circle of bay leaves framing the whole device, which had evidently been recently prepared for the occasion. Salutes were fired in London and in many other places both at home and abroad, and several dinners were held in the Queen's honour. The Mayor of Westminster, at a dinner to the Westminster City Council, proposed "The Queen," and alluded to the fact "that by her industry, frugality, and affectionate regard for all classes of the people, by the interest she had displayed in the welfare of the soldiers, who in South Africa were fighting for their King and country, by her association with the nurses of the country and in other ways, she had endeared herself to the hearts of the people. They were grateful to Almighty God for having given the King such a consort. Long might she be spared, and long might her example be followed by the women of England." And all nurses will echo with fervour the words of Lieutenant-Colonel Clifford Probyn.

THE winter sale of the handicraft of the Working Ladies' Guild was held this week in the Indian Museum at 24 Park Lane, the residence of Lord and Lady Brassey. The room itself is wonderful, the walls from floor to roof being a mass of Indian carved woodwork, and it made an extremely appropriate setting for all the beautiful work which was exhibited. The object of the Guild is to provide employment for those ladies in need of it, and this is the twenty-fourth year that it has been able to render real assistance to between 60 and 70 ladies. Not only was art needlework and plain needlework on view on Tuesday, but the specimens of lacquered leather work in imitation of the old Spanish Cordova lacquered leather work of the fifteenth century were most interesting, as were also the embroidered covers of the books. These working ladies are making a speciality of doing up old furniture, more particularly regilding and repairing tapestry. The sale was opened by Princess Beatrice, who presided over one stall, selling goods and receiving money in the most pleasant businesslike manner. A cover, which was a copy of the binding of a book belonging to Anne Boleyn, was almost her first sale, though she had no lack of buyers during the whole of the time she remained.

LORD MILNER seems to have gained a warm place in the hearts of the South African people. From a letter received this week I learn that he lately paid a private visit to one of the British refugee camps. After often waiting weary months, a good many of the refugees have got passes back to the Rand, but a large number still remain, and some of the local authorities were very anxious that the High Commissioner should see the contrast between the comfort and excellent management of the Boer concentration camps and the rough and ready way in which our fellow countrymen have to live. Lord Milner seemed much impressed, and promised to help in the matter. The genuine kindness he manifested went far to cheer the poor refugees, and one woman ran out of her tent and begged him to come and see her garden. In the midst of the wilderness of sand she had managed to get together quite a bright little array of flowers, which she had protected and sheltered by surrounding them with paraffin tins, odd boxes, or anything else she could find. The High Commissioner at once acceded, passing through the woman's tent to reach the garden, and much pleased the amateur gardener by his praise.

ONE word of warning. I so often see queries from nurses and others anxious to go out to South Africa, but every private letter received emphasises the fact that the time is not yet. It is very difficult to obtain permits even to land from the steamers now, and those who get ashore find accommodation very scarce, living very dear, and work not to be had. People who own land in Durban and care to sell are making money hand over hand, but that tends to keep the price of everything else up, and there is still only one word for those who wish ultimately to go to South Africa, "Wait."

PARTISANS had looked forward to the Devonian dinner at the Hotel Cecil on Saturday last as an occasion when the guest of the evening would be able to speak without restraint, and would, in consequence, give the Government some hard knocks. But to the disappointment of the sensation-mongers, and to the delight of those who are the real friends of the reliever of Ladysmith, Sir Redvers Buller made an excellent speech, thanked not only the Devonians but the world at large for their much valued sympathy, and told no one anything. He managed, however, to impress upon his hearers the fact that he knew too much about the difficulties of fighting in South Africa to have any sympathy with those who say that the war ought to be over, and he spoke warmly of the 200,000 troops who are now doing "their level best" to bring it to a "definite, final, downright conclusion." The affection of the masses—in many cases the juvenile and unreasoning masses, children being largely represented—for General Buller was shown earlier in the week when he was recognised at Ludgate Circus and cheered vociferously, and on Sunday when there was a mass meeting in Hyde Park, with four platforms, banners, and shouting and wholesale abuse of the Government.

EARL GREY, who is now so well known as the pioneer of the movement for the reform of public-houses, is troubled at the physical deterioration apparent in the young Englishmen of the lower classes. According to the recruiting returns at Manchester, out of every 11,000 men volunteering for service in South Africa 8,000 were rejected as unfit to carry a rifle on the veldt, and out of the 3,000 accepted further examination proved that about 1,200 only came up to the recognised standard of what a soldier ought to be. With the object of finding out how far the stunting power which, it is believed, town life exercises upon the physical frame is of a serious nature, Lord Grey suggests that all school children should be weighed and measured twice a year—Parliament granting special powers—so that the conditions under which children best develop into healthy men and women may be ascertained. All will, I think, admit that much useful information may be obtained in this way, but the practical remedy is more difficult to discern. How can fresh breezes be provided for children suffering from the air of the slums, or more food for the little ones of ill-paid parents? The only remedy, at first sight, seems adoption by the Government at birth of every child likely to be reared in unhealthy surroundings, and that, of course, is an impossibility.

LAST week I mentioned that the memorial service for the soldiers who have fallen in South Africa during the year would be held in St. Paul's Cathedral on Monday, December 30. The date has since been altered to Monday, December 16. The hour is 3 P.M., and most of the Cathedral will be open to the public.

### A Book and its Story.

"THE INDEPENDENT WOMAN."*

In "The Benefactress" the author of "Elizabeth and Her German Garden" and that even more delightful book, "A Solitary Summer," leaves her communings with Nature, and presents to her readers some subtle character studies. As before, the main interest of this new story is centred in the country of her adoption. Into the sordid environment of a German farm homestead, of the type which in England finds its counterpart in the snug homeliness of a small country estate, with cultivated outdoor surroundings and ordered refinement within, she transports her heroine, Anna Estcourt.

"When Anna Estcourt was 25, she had begun to wonder whether the pleasure extractable from life at all counterbalanced the bother of it. Many and various are the motives that impel a woman so to ponder. In Anna's case the motive was nothing more exalted than the perpetual presence of a sister-in-law. The sister-in-law was rich—a pleasing circumstance; but the sister-in-law was also frank, and her husband and Anna were entirely dependent on her, and her richness and frankness combined, urged her to make fatiguingly frequent allusions to the Estcourt poverty. Except for their bad taste her husband did not mind these allusions much, for he considered that he had given her a full equivalent for her money in bestowing his name on a person who had practically none; he was Sir Peter Estcourt, of the Devonshire Estcourts, and she was a Dobbs, of Birmingham. Besides, he was a philosopher, and philosophers mind nothing." Anna, not being a philosopher, minded many things; above all, the fact of being unable from lack of means to be independent of the suffrages of her wealthy sister-in-law. Marriage was the only solution of the difficulty, and marriage was not the height of Anna's ambition. Of suitors she had no dearth; perhaps the daily contemplation of an ill-matched pair made her naturally anxious to avoid an alliance in which, while material comfort abounded, her heart would be starved. No; not happiness, or so-called happiness, she craved. Only to be able to add some little drop of sweetness to the bitter cup of other lives, that was all she asked, reading an exaggerated meaning, perhaps, into the sorrows which, out of the bitterness of her own soul, she saw in the face of the world around her.

"Oh, if I could, I would comfort everyone that is sad or sick at heart and sorry—if I could!" At that moment, from a curiously unexpected and incongruous incident, the sudden arrival of a German uncle, known to her as Uncle Joachim, came indirectly the golden key that enabled her to unlock the door of that future for which she longed—a future in which self would be lost in the service of those endowed through her munificence.

Uncle Joachim had come—not, as he had told Susie (the sister-in-law), to see London, but to see Anna herself, and to find out how it was that "this pretty creature, so soft and young, so lovable," should have reached an age that in Germany is the age of old maids, without marrying. Anna was delighted to have him with her; they went frugally to bakers' shops and ate buns; they did not take hansoms, for Uncle Joachim was thrifty. But the old man was a willing victim to Anna's charm, and very soon had drawn her out, until he saw, in spite of an apparently unobservant manner, where lay the trials that were daily fretting her. They went everywhere together—to service at St. Paul's, among other places. She felt with happy conviction that blood was thicker than water, after all, and she realised vaguely that he sympathised and understood. It was only after a service there that she began to think there might, in spite of these reflections, be points of taste on which they differed. "He slept through the anthem, one of those unaccompanied anthems that are sung there with what seem of a certainty to be the voices of angels." And in coming out, when a fugue was rolling in glorious confusion down the echoing aisles, and Anna, who preferred her fugues confused, felt that her spirit was being caught up to heaven, he had looked at her rapt face and wet eyelashes, and patted her hand and said very encouragingly, "In my youth I cultivated Bach; now I cultivate pigs. Pigs are better."

The old man died soon after his return to Germany. Anna received a letter, written shortly before, in which he said that as she appeared to be possessed of an obstinacy more becoming in a man than a woman, whose calling it was to be gentle and yielding, and convinced from what he had seen that she could never be happy with her sister-in-law, and, also because, "it was beneath the dignity of his sister's daughter, a young lady of good family, for ever to roll herself in the feathers with which the middle-class goose, born Dobbs, had furnished Peter's otherwise defective nest," he had decided to make her independent altogether by bestowing upon her the only property he could leave to whomsoever he chose, a small estate near Stralsund. Here Anna would have the opportunity of leading the better life, which was always, he added, "a life of simplicity, frugality, and hard work." With this letter came one from Uncle Joachim's solicitor, saying that his instructions were to forward it to Anna after her uncle's death. Anna wasted no time in fulfilling her uncle's wishes. She started immediately for Germany, accompanied by her sister-in-law and her daughter and governess. In the delineation of German character the authoress is specially happy, and when Anna arrives at the remote little town in Pomerania adjacent to her estate we feel that the real business of the story has begun. From that hour Anna appears in a new light. She receives patiently the various deputations that greet her progress until the door of her new domicile is reached. Susie, her sister-in-law, does not share her optimism, and terms the "park" as "a horrid, gloomy, damp wilderness." The low, white, two-storeyed house, separated from the forest by a circular grass-plot and a ditch with half-melted snow in it and muddy water—a house quite by itself among the creaking pines, with a great many windows and a brown-tiled roof—was the home bestowed by Uncle Joachim on his dear and only niece Anna. Here Anna decides to remain, and to make the house beautiful within for the reception of "twelve homeless ladies of good family." She engages a German Princess as chaperone and house-director, and then sets to work with energy for the reception of her guests. When the house is ready, an advertisement is inserted in one or two leading newspapers. The impression, of course, of those to whom she speaks of her scheme is that she, like all English people, is a little mad.. The "Frau pastor" is lost in wonder. "The cost will be colossal," she said, surveying Anna from head to foot; "but then, the gracious Miss is rich."

Anna's philanthropic designs are not quite as successful as she dreamed they would be. She finds herself encircled by a network of suspicion and intrigue on the one hand, and ingratitude and distrust on the other. The leading of the better life under these circumstances is difficult, and Anna realises that perhaps one of Uncle Joachim's first suggestions was, after all, the true solution of her difficulties. "Independence is not a word for the lips of women; it is a woman's pride to lean on a good husband, to be shielded and protected by him." She follows it later, and finds happiness and peace of mind in the holy estate.

* (By the author of "Elizabeth and Her German Garden." One vol. 6s. Publishers; Macmillan.)

### Notes and Queries.

**The Editor is always willing to answer in this column, without any fee, all reasonable questions, as soon as possible.**
**But the following rules must be carefully observed :—**
**1. Every communication must be accompanied by the name and address of the writer.**
**2. The question must always bear upon nursing, directly or indirectly.**
**If an answer is required by letter a fee of half-a-crown must be enclosed with the note containing the inquiry.**

*Travelling Expenses.*

(93) Will you kindly tell me if it is customary for a district nurse to have her travelling expenses, when entering on her duties, paid by the committee engaging her ?—*J. S.*

This is entirely a matter for private arrangement between the contracting parties.

*Ringworm.*

(94) Can you tell me where Poor Law children, suffering from ringworm, would be taken at a weekly payment?—*G. A. W.*

You might apply at the Hospital for Diseases of the Skin, 52 Stamford Street, Blackfriars, S.E., and to the Western Skin Hospital, 179 Great Portland Street, W.

*Homes.*

(95) Can you tell me of a home in the North of England for a male patient suffering from abscess on the brain? He is bedridden, blind, and has some delirium. His friends could pay a small sum weekly for his maintenance.—*E. F.*

The Home for Incurables for the Border Counties, Strathclyde House, Carlisle; the Home for Incurables, Spital Tongues, Newcastle-on-Tyne; and Christ's Hospital, Sherburn, near Durham, appear the most likely to receive the case. Apply to the secretaries. But why not a hospital if it is really an abcess ?

Can you tell me of a Home of Rest where a lady suffering from spinal concussion could be received for some months at from 12s. 6d. to 15s. 6d. a week ?—*Spinal Concussion.*

Either St. Peter's Memorial Home, Maybury Hill, Woking, or the Convalescent Home for Women and Children, New Brighton, Cheshire, seem to meet the requirements of the case. See "Burdett's Hospitals and Charities" for fuller lists.

Will you kindly tell me if there is any seaside home for patients suffering from spinal disease ?—*M. C.*

You give no particulars. See "Burdett's Hospitals and Charities," as you may find something suitable under the heading "Chronic and Incurable."

Will you kindly tell me of a home where a married man, 23 years of age, could be sent? He is going blind from constitutional weakness.—*Nurse Eliz. K.*

There are a large number of charities for the blind, both in London and the provinces, for which consult "Burdett's Hospitals and Charities." You could see a copy at this office. But if he is "going blind" why not an ophthalmic hospital ?

Will you kindly tell me of a home suitable for a working woman, where the Salisbury treatment is carried out ?—*Grace M.*

This treatment is usually carried out at private institutions at which the fees are comparatively heavy.

Can you tell me of a home for a girl of eight? She is quite helpless, and unconscious of all she does. Her parents can afford to pay a small sum weekly.—*N. M.* (Liverpool).

If the case is capable of improvement it might be admitted to the Royal Albert Asylum and Training Institution for the Feeble-minded of the Northern Counties, Lancaster. If not, try St. Elizabeth's Home, Mortimer Street, London, W.

Can you tell me of a home where a girl of sixteen, mentally deficient but quite harmless, might be trained ?—*Mrs. H.*

Apply to the Secretary of the National Association for Promoting the Welfare of the Feeble-minded, 53 Victoria Street, S.W., for advice.

Can you tell me of a home, not expensive, in Scotland, or England, where a nurse addicted to laudanum drinking could be received for treatment.—*J. B.*

Such homes are private as a rule, and are therefore only reached by personal recommendations, or by advertisement.

Can you tell me of a comfortable home where a man, aged 74, suffering from locomotar ataxia could be taken. He could pay 10s. or 12s. a week.—*Nurse S.*

The Hospital for Epilepsy and Paralysis, 32 Portland Terrace, Regent's Park, N.W., receives a few private patients of this kind.

Can you recommend any free home where an elderly woman, who has been a ladies' nurse, could be received for the winter? She is partly incapacitated by rheumatism.—*J. B. H.*

The Royal Mineral Water Hospital, Bath, is open to the poor of the United Kingdom upon the production of a medical certificate and a certificate of poverty. This might suit.

1. Will you kindly tell me of a home for inebriates (female) in or near Yorkshire. 2. Also if, having the consent of a doctor and friend, must a paper be signed by the patient herself ?—*S. J.*

1. The High Flatts Sanatorium for Women, Denby Dale, Huddersfield (in connection with the Yorkshire Women's Temperance Union) might receive her. 2. Yes.

Will you kindly tell me of a home for a little boy of 10. He has apoplectic seizures, and is slightly imbecile. His parents can pay a small amount.—*Nurse R.*

See reply to *N. M.* and *Mrs. H.*

Would you kindly tell me whether there is any home which would take a lady suffering from cancer ? She is able to get up during the day.—*A. F.*

The Cancer Hospital, Fulham Road, Brompton, S.W., or the Middlesex Hospital, Mortimer Street, might receive the case.

Is there any institution where a young girl mentally afflicted, and who is now an inmate of the county asylum, could be received? She is passive, and gives no trouble, but requires watching in case she should ramble away. The reason we wish to remove her is, that the surroundings appear to depress her, and it is feared this may retard her recovery. Our means are very limited, but we would do our best towards paying cost of maintenance.—*J. C.*

See reply to *Mrs H.*

Would you kindly tell me of a home where a delicate infant could be received for a small sum weekly? The mother is in hospital and her recovery is very doubtful.—*Nurse B.*

Apply to Dr. Barnardo, Stepney Causeway, E.

*Private Nursing Association.*

(96) Can you give me the address of the association of private nurses of which Mrs. Bedford Fenwick is the head ?—*Anon.*

The Registered Nurses' Society, 269 Regent Street, W.

*Varicose Veins.*

(97) I have suffered for many years from varicose veins in both legs and am wearing rubber bandages, but there is now appearing one large vein running down the inside of the right thigh. I have heard that something can be done by strapping with plaister to relieve. Will you kindly tell me if this is practicable, and, if so, what kind of plaister is suitable ?—*Veritas.*

This is evidently a case for surgical advice. As age advances varicose veins in the leg become a serious matter, and the question of removing the vein is one which might require consideration.

*Text-Book on the Use of Roentgen Rays in Hospital Work.*

(98) Could you kindly tell me the name and publisher of some inexpensive text-book relating to the practical use of radiography in hospital work, which would also give the technique of the development of the radiograph exposures. They have an X-ray plant at a hospital I am going to work at, and it is desirable for the nurses to have some knowledge of the working of it ?—"*Casualty Pro.*"

"Practical X-ray Work." By Frank T. Addyman. (Scott Greenwood and Co. 10s. 6d.)

*Erysipelas Patients.*

(99) I should be glad to know how masks of lead lotion are kept in place upon erysipelas patients. Whether they are bandaged on and oil paper applied, or simply the lotion upon lint kept in place by tape attached to the mask ?—*A Reader.*

If you have been trained, you must know how this is done, and that much must depend upon the place where they are applied.

*Manicure.*

(100) I am thinking of taking up manicure as a living, but I cannot find out where proper lessons are given, nor the terms. Are there any books published on this work ?—*C. J. W.*

This query does not appertain to nursing. So many nurses, however, appear interested in the subject that, having made inquiries, we are able to give the following particulars. Manicure requires great dexterity and long practice as well as a special aptitude for it. Generally an assistant showing promise is carefully drilled for two or three years, after which she is allowed to attend to a customer. Ladies' maids, etc., are at some establishments permitted to take a course of lessons, the usual price being £1 1s. for six. Ladies intending to go in thoroughly for the art would be required to pay premiums and to serve fitting apprenticeships. A few lessons will not make a manicurist.

**Standard Books of Reference.**

"The Nursing Profession: How and Where to Train." 2s. net; post free 2s. 4d.
"Burdett's Official Nursing Directory." 3s. net; post free, 3s. 4d.
"Burdett's Hospitals and Charities." 5s.
"The Nurses' Dictionary of Medical Terms." 2s.
"Burdett's Series of Nursing Text-Books." 1s. each.
"A Handbook for Nurses." (Illustrated). 5s.
"Nursing: Its Theory and Practice." New Edition. 3s. 6d.
"Helps in Sickness and to Health." Fifteenth Thousand. 5s.
"The Physiological Feeding of Infants." 1s.
"The Physiological Nursery Chart." 1s.; post free, 1s. 3d.
"Hospital Expenditure: The Commissariat." 2s. 6d.

All these are published by the Scientific Press, Ltd., and may be obtained through any bookseller or direct from the publishers 28 and 29 Southampton Street, London, W.C.

### Travel Notes.

By Our Travelling Correspondent.

### LXXXVII.—THE GREAT MASTERS OF THE PAST.

The kind interest taken in my gossiping papers on Rome last winter has encouraged me to continue the same subject during the dark, cold weeks of December and January, and to introduce from time to time short notices of those great artists of the past whose works furnish so much of the pleasure of our modern Continental travel.

To know something, however slight, of the life history of these giants in art adds greatly to our enjoyment as we stand before their wondrous works, and makes them living creatures to us rather than dull names and numbers in a catalogue.

As we gaze upon Andrea del Sarto's sweet "Madonna with the Christ and St. John," we think of the harassed artist, who loved not wisely but too well, and was compelled to work to the destruction of health to keep roof over the head of his worthless and extravagant wife. In looking at Cellini's masterpieces we think of the gay and far from respectable ruffler who swaggered up and down the Ponte Vecchio, ever ready to pick a quarrel upon the smallest provocation, or indeed with no provocation at all, but simply to prove himself the better man—frequently in prison upon the most disreputable charges, and between whiles bandying insults with his hated rival, Bandinello. Or again, as we stand before the gentle, pale-faced mothers in the pictures of the monk Fra Filippo Lippi, does not his strong face rise before us, telling its own plain story of a disposition ill suited to the cloister? Restless, loving, and wildly daring, he broke all vows and stole away the woman he loved one dim, misty morning from her convent at Prato. To-day I propose to glance for a few moments at the life of Donato di Belto di Bardi, or, as we are more accustomed to call him, Donatello, whose amiable character, generous disposition and purity of life at a time when manners and morals in Florence were most corrupt endears his memory to us.

### "Our Own Little Donatello!"

So to this day do the art-loving Florentines speak of Donato, though he has been dead more than four hundred years, and was at the time of his death a very aged man.

He was born at Florence in 1383, and must have early shown some feeling for art, for we find him as a child pursuing his studies in that line under the patronage of the Martelli family, to whom throughout his life he testified the most grateful affection.

The first work of his which made a mark was an "Annunciation," which he carved in Macigno stone for the chapel of the Cavalcanti in Santa Croce. This brought him under the notice of Cosimo de Medici, who remained always his firm friend and supporter.

### The Rival Crucifixes.

His next work of importance, and one which proved a turning-point in his artistic career, was a crucifix in wood, and of this episode Vasari gives us a delightful account. Having accomplished the work, he invited the criticism of Brunelleschi, who found serious fault with the figure, affirming it was neither more nor less than a peasant, with no attributes of divinity. The young Donato, mortified and disappointed, exclaimed petulantly that it was no easy matter to work in wood, and that since Brunelleschi thought so poorly of his work he had better try if he could improve upon it. The elder artist accepted the challenge, and said nothing till the work was complete, when meeting Donatello in the city, in the wonderful old Mercato Vecchio (alas! now no longer existing), he invited him to dinner in his studio. He then bought provisions, stowed them in his guest's sculptor's apron, and asked him to go to his house, where he would shortly join him. As Donato entered his friend's workroom his eye was caught by the magnificent crucifix. Exclaiming "Lord! what form! what sublimity!" he let go his apron, and all the contents fell to the ground, consisting, as our old friend Vasari (always eloquent on details) tells us, of eggs, butter, etc. Brunelleschi, entering at the moment, affected to see nothing, and invited his friend to table; but Donatello, overcome by generous admiration, exclaimed, "Nay! I am not worthy to eat with you—I the portrayer of boors—you the sculptor of gods!"

It is a pretty and natural story, and is commonly repeated to this day among the populace of Florence. In the chapel of the Cavalcanti at Santa Croce there still hangs the once-despised crucifix; and in Santa Maria Novella, in the chapel to the left of the choir, one sees Brunelleschi's masterpiece. Donatello was right; it is a god-like figure.

### His St. George, St. Mark, and the Bald Man.

This severe lesson was not barren of result on the young Florentine; his style gained rapidly in breadth, grace, and nobility, without losing its originality and simplicity. In proof of this, look at his St. Mark and St. George on the outside of that architectural gem Or San Michele. The real statues are now in the Bargello, and exact replicas are placed in the niches of the church. One cannot but regret this on sentimental grounds, though doubtless, considering the priceless value of these treasures, it is well that they should be removed from the attentions of the hordes of Florentine boys swarming about the Street of the Stocking-makers. Of the St. Mark, Michael Angelo is reported to have said it would have been impossible to reject the Gospel of such a straightforward man. Of all Donatello's works, nothing affects me so much as his St. George The various city guilds each presented the figure of a saint to the church—that of St. Mark represented the furriers, and that of St. George the armourers. What a lovely figure it is! Brave, simple, pure and dignified, it seems to show us what true religion and courage can do to overcome the difficulties and temptations of this world, so lavishly spread before the eyes of young manhood.

Donatello himself considered the portrait of Barduccio Cherichini, known on account of his baldness as "the Zuccone," to be one of his best works, and sitting before it he was heard to say, "Speak, friend; speak!" And often he used it as a form of attestation, thus: "Sulla fè che io porto al mio zuccone" ("On the faith I have in my bald man"). At the age of eighty-three, full of years and honour, Donatello died in the Street of the Melon in the year 1466, tenderly cared for by the Medici to the last.

### TRAVEL NOTES AND QUERIES.

Rules in Regard to Correspondence for this Section — All questioners must use a pseudonym for publication, but the communication must also bear the writer's own name and address as well, which will be regarded as confidential. All such communications to be addressed "Travel Editor, 'Nursing Section of *The Hospital*,' 28 Southampton Street, Strand." No charge will be made for inserting and answering questions in the inquiry column, and all will be answered in rotation as space permits. If an answer by letter is required, a stamped and addressed envelope must be enclosed, together with 2s. 6d., which fee will be devoted to the objects of "The Hospital" Convalescent Fund. Any inquiries reaching the office after Monday cannot be answered in "The Hospital" of the current week.

Venice in the Spring (Potsdam).—It will be too early at the time you mention, but why not travel *viâ* the Brenner, by Cologne, Munich, and Innsbruck, remaining at Innsbruck, which is a nice winter place, until the first week of April. If that month is cold, defer Venice still later, for it can be extremely cold there, because the sun is shut out by the tall houses, and the side canals are narrow and dark. For a long stay I should advise some hotel on the Rio a degli Schiavoni, as being more airy and open. Yes, the Danielli is very pleasant, but rather dear.

Lisieux *versus* Rouen (Poppy).—Both are charming from an artistic point of view, though Rouen is terribly "restored," but Lisieux is a comparatively small place, and you might get tired of it for a prolonged stay. At Rouen, when the weather is fine, you can make charming excursions in the neigbourhood, and when it is bad, as you intend to do interiors, there is rich wealth for your brush in the many churches.

